# Half-release and full aspiration technique for endoscopic ultrasound-guided hepaticogastrostomy in severe acute cholangitis

**DOI:** 10.1055/a-2860-7515

**Published:** 2026-05-19

**Authors:** Shunsuke Omoto, Mamoru Takenaka, Akane Hara, Yasuo Otsuka, Akihiro Yoshida, Kosuke Minaga, Masatoshi Kudo

**Affiliations:** 1 Department of Gastroenterology and HepatologyKindai University Faculty of MedicineSakaiJapan

Endoscopic ultrasound (EUS)-guided biliary drainage is an established alternative to endoscopic retrograde cholangiopancreatography (ERCP) in patients with inaccessible papilla 1 . In severe acute cholangitis, intrabiliary pressure is markedly elevated and further raised by contrast injection, increasing the risk of bile leakage and peritonitis through the immature fistula 2 3 . Based on the full aspiration technique for EUS-guided pancreatic fluid drainage 4 , we describe a “half-release and full aspiration” technique during EUS-guided hepaticogastrostomy (EUS-HGS) for complete bile aspiration to prevent bile peritonitis ( Video 1 ).

EUS-guided biliary drainage in severe acute cholangitis after partial duodenectomy. After half-release of the Type IT stent, the guidewire was removed and 40 mL of bile was aspirated through the delivery system lumen before final stent release. EUS, endoscopic ultrasound.Video 1

**Fig. 1:**
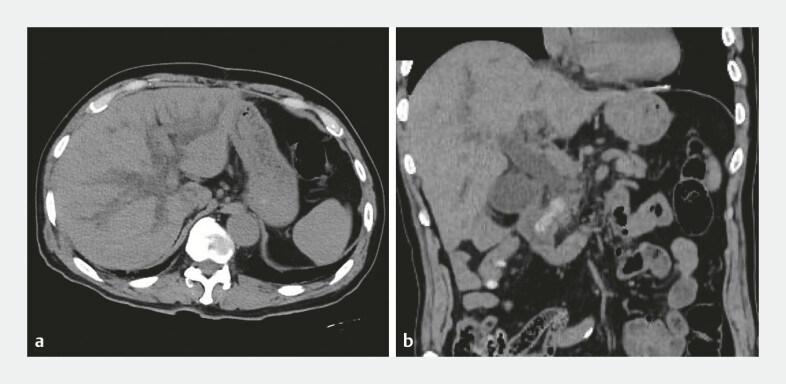
Pre-procedural computed tomographic images. **a** An axial image showing marked intrahepatic bile duct dilatation. **b** A coronal image showing choledocholithiasis, with multiple stones impacted in the distal bile duct.

An 82-year-old man who had undergone partial duodenectomy presented with severe acute cholangitis (Grade III, TG18 5 ) due to choledocholithiasis with transient altered consciousness. Double-balloon enteroscopy-assisted ERCP was attempted, but biliary cannulation failed due to marked respiratory motion that prevented a stable view of the papilla. EUS-HGS was performed as an alternative approach ( Fig. 1 ).

**Fig. 2:**
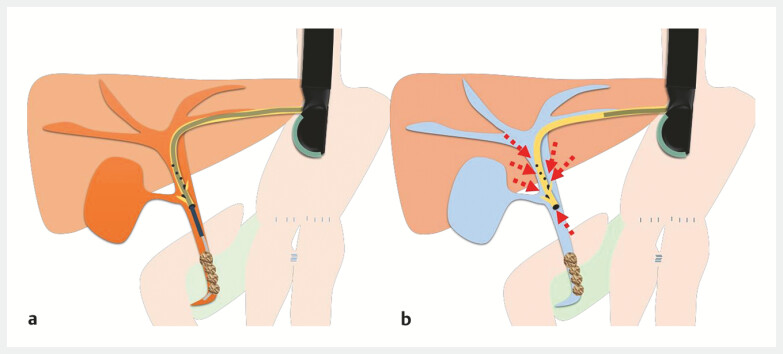
Endoscopic placement of a plastic stent into the bile duct. **a** Advancement of the plastic stent catheter into the bile duct over the guidewire. **b** Half-release of the plastic stent, followed by guidewire removal and full bile aspiration through the inner guiding catheter lumen before final stent deployment.

**Fig. 3:**
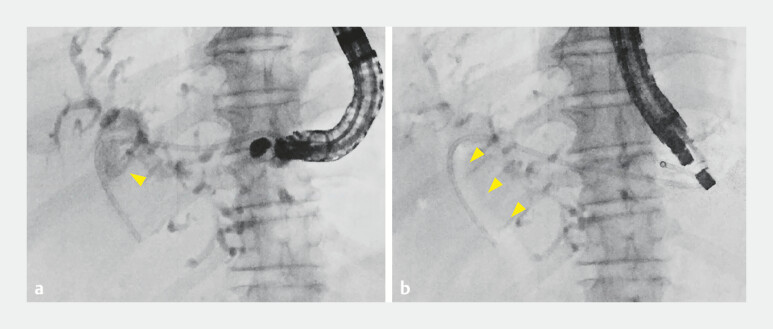
Fluoroscopic images obtained during the procedure. **a** Contrast medium filling the bile duct lumen. **b** Pneumobilia in the common bile duct immediately after plastic stent placement, indicating successful biliary decompression.

**Fig. 4:**
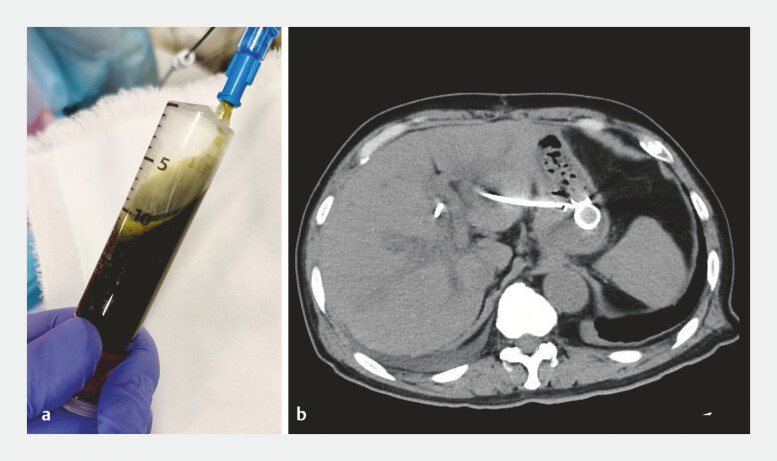
Intra- and post-procedural images. **a** Aspiration of approximately 40 mL of bile through the inner guiding catheter lumen. **b** A post-procedural computed tomographic image showing appropriate stent placement, resolution of biliary dilatation, and no evidence of biliary peritonitis.

The intrahepatic bile duct was punctured with a 19-gauge needle (Areus; Century Medical, Tokyo, Japan). A 0.025-inch guidewire (VisiGlide 2; Olympus) was advanced, and tract dilation was performed using a drill dilator (Tornus ES; Olympus). To allow bile aspiration through the delivery system lumen, a dedicated plastic stent for EUS-HGS (Through and Pass, TYPE-IT; Gadelius Medical Co., Ltd, Tokyo, Japan) was half released. The guidewire was then removed, and bile was fully aspirated through the inner guiding catheter lumen ( Fig. 2 ). After stent deployment, pneumobilia was subsequently observed in the bile duct ( Fig. 3 ). A total of 40 mL of bile was aspirated, and post-procedural computed tomography confirmed appropriate stent placement ( Fig. 4 ). Complete bile aspiration before stent deployment reduces intrabiliary pressure and may prevent bile peritonitis. This approach may improve the outcomes of EUS-HGS using dedicated plastic stents.

Endoscopy_UCTN_Code_TTT_1AS_2AK
